# Study of Clinical Results and Functional Outcome of Patients With Distal Femur Fracture Treated With Dual Plating

**DOI:** 10.7759/cureus.34182

**Published:** 2023-01-25

**Authors:** Mukund Pai Manjeswar, Amit Kale, Harsh Raithatha, Shail Shah

**Affiliations:** 1 Orthopaedics, Dr. D.Y. (Dnyandeo Yashwantrao) Patil Medical College and Hospital, Pune, IND

**Keywords:** medial assisted plating, functional and clinical outcome, dual plating, fracture, distal femur

## Abstract

Introduction

Distal femur fracture has been routinely fixed with a single lateral locking plate. This method of fixation in intra-articular distal femur fractures has proved to give a higher outcome of varus collapse as well as higher rates of mal-union due to inadequate fixation of the medial aspect of the distal femur. To address this drawback of single lateral plating, the use of medial assisted plating (MAP) has been introduced recently, which was proposed to give better stability to the medial fragments. This Is a prospective case series of 50 patients with distal femur fractures treated with dual plating.

Materials and methods

Fifty cases of patients with distal femur fractures were treated with dual plating between August 2020 and September 2022. Patients were followed up postoperatively till the third month, when patients were assessed clinically and radiologically. Range of motion of the knee, postoperative fracture displacement, limb shortening, and signs of union and infection were checked. Neer's scoring and Kolmet's scoring were used to grade the outcome for the patients.

Results

The mean age of the patients was 39. Only 12% of the cases were open fractures. Eighty-four percent of the cases did not have fixed flexion deformity (FFD) and only 4% had FFD of 15 degrees; 72% of the cases achieved flexion of the knee beyond 120 degrees. Eighty-four percent of patients had normal walking ability by the 12th week postoperatively; 16% of the cases had a postoperative displacement of more than 1.6 cm, with the maximum being 2.5 cm.

Conclusion

From the study, we have concluded that outcomes were better for fractures of distal femur when treated with dual fixation, probably due to superior fixation and earlier postoperative mobilization.

## Introduction

Fractures of distal femur are rare, accounting for less than 0.5% of total fractures and contributing 3-6% of all femoral fractures with a bimodal pattern distribution of with peak in frequencies found in elderly females in their late 70s and young males in their 30s, with males having a history of high-velocity impact trauma and the females having lower energy trauma with osteoporosis [1,2]. Distal femur fractures were traditionally managed utilizing the principle of Watson Jones and John Charnley, which included skeletal traction, closed reduction of the fracture, and casting [3,4]. Poor outcomes of conservative management led to the need for surgical intervention, which comprised using various modalities of fixation such as condylar buttress plates, dynamic condylar screw fixation, locking compression plates, fixed angle condylar blade plates, and retrograde interlocking nails [5]. The SoFCOT (Société française de chirurgie orthopédique et traumatologique) symposium of 1998 reported infection and septic non-union in 13%, aspetic non-union in 14%, residual knee stiffness in 35%, post-traumatic secondary osteoarthritis in 30%, with loss in reduction and chondral damage [6]. Locking plates provided higher levels of stability and resistance to implant failure as compared to retrograde nails [7]. Recent biomechanical studies suggest better implant stability and reliability of distal femur locking compression plate (LCP) compared to retrograde nails [8]. Currently, minimally invasive plate osteosynthesis (MIPO) and less invasive stabilisation system (LISS) procedures have been developed which help in achieving better anatomical fixation while also reducing soft tissue damage during the process of fixation [5]. Adequate rigid fixation of distal femur fractures with minimal soft tissue damage and preservation of blood supply along with appropriate physiotherapy can attain better clinical outcomes and facilitate earlier weight bearing [9]. Complications associated with this fracture fixation with plating include varus collapse, non-union, arthrofibrosis, restriction of knee range of motion, and infection [10]. Single lateral locking plates would fail in distal femur fractures with gross metaphyseal comminution, medial cortical defects, or bone loss, and in osteoporotic fractures. Such fractures require additional medial plating to buttress the medial column [11]. 

In the study by Chi-Chuan et al. in 2007, where they assessed femoral supracondylar malunions with varus medial condyle and shortening, it was noted that in these fracture patterns, a retrograde nail usually can not be used, as inserting a retrograde nail from the intercondylar notch cannot correct the malalignment [12]. Single-stage antegrade locking intramedullary nailing was noted to be an effective technique for patients with femoral supracondylar malunions with varus medial condyle and shortening. They proposed that medial buttress plating is complicated because it is difficult to contour the medial plate after osteotomy and proximity of the femoral artery [12]. In the study done by Salas et al. in 2015, where locking plate and retrograde intramedullary nail fixation were compared biomechanically in osteoporotic bones, it was found that the probability of fracture healing (POF) was higher in locking compression plating as compared to intramedullary nailing, 21.8% vs 0.019%, under applied loading conditions [13]. In the 2016 study by Briffa et al., where biomechanical analysis was done to compare the stability of medial-assisted plating (MAP) with single lateral locking plating in axial loading, medial plating showed a significantly decreased displacement, bending moment and strain at the fracture site during axial loading. The study concluded that medial plating of a comminuted supracondylar femur fracture is a more stable construct than single lateral plating [14]. In a study done by Shroff et al. in 2017, LCP had better results as compared to supracondylar nailing (SCN) in all parameters, especially used for intra-articular fractures. Results were graded according to Schatzker and Lambert's criteria. In the LCP group, 28 (62.22%) had excellent results, 12 (26.66%) had good, two (4.44%) had fair, and three (6.66%) had poor, while in the SCN group, 22 had excellent results (48.88%), nine had good (20%), six had fair (13.33%), and eight had poor (17.77%) results [15]. 

In the study by Rongbin et al. in 2018, the clinical efficacy of augmented MAP for treating comminuted metaphyseal fracture of distal femur, they compared the operation time, intraoperative blood loss, fracture healing time, and postoperative knee function recovery between two groups; one comprising of patients with distal femur fractures treated with single distal lateral locking plate and the other group was treated with MAP. Besides the longer intraoperative time, the outcome was significantly better in the group that had MAP, with an increase in stability of comminuted fracture and improved fracture healing rate (average healing time of 5.39 ± 0.69 months). A total of 90.9% of the dual plating cases had excellent and good outcomes [16]. In the 2018 study by Bai et al., MAP was compared with the traditional single lateral locking plating for distal femur fractures. The outcome in terms of Kolmert’s score was better in MAP; 33% of cases had excellent outcomes in MAP as compared to 31% in single lateral plating [17]. In the 2019 study by PArk et al., a biomechanical assessment was done in the additional fixation of medial plate over unstable lateral locked plating of distal femur fractures. It was noted that all single lateral locked plates showed plate bending at the fracture gap, while this did not occur in dual-plating cases. Load to failure was 17.1% greater in dual plating cases as compared to single lateral plating. Postoperative displacement was a mean of 5.6 mm in dual plating while it was 8.8-9.1 mm in single lateral plating. It was concluded that addition of medial plate in distal femur fracture fixation increased fracture stability [18]. In the study done by Rollick et al. in 2020, where the effect of dual plating on the vascularity of distal femur was observed, they reported that lateral locking plate application resulted in a mean of 21.2% reduction in distal femur vascularity. The addition of medial plate did not markedly decrease the vascularity, hence attributing the majority of the reduction in vascularity to the lateral plating. Hence, the study proposed the consideration of medial plating in the setting of comminution and poor bone stock in distal femoral fractures [19]. 

## Materials and methods

This is a prospective study done with the approval of the Ethics Committee of Dr. D. Y. Patil Medical College, Hospital & Research Centre, Pune, Maharashtra, India (Approval number: IESC/PGS/2020/93, dated March 24, 2021. The study included 50 patients with distal femur fractures between August 2020 and September 2022, who were admitted to the orthopaedics and trauma department of Dr. D. Y. Patil Medical College, Hospital & Research Centre. Informed and written consent was taken for all patients who were included in the study. 

Inclusion and exclusion criteria

The following are the inclusion criteria: (i) Patients aged ≥ 18 years old, (ii) Patients aged < 80 years age, (iii) Recent trauma leading to fractures of distal femur, (iv) Closed fractures of distal femur, and (v) Open fractures of distal femur till Gustilo Anderson type 3B.

The following are the exclusion criteria: (i) Previous knee trauma or surgery, (ii) Pathological fractures, (iii) Spine injury and neurological deficits, (iv) Neglected old fractures (more than 21 days after onset of trauma), and (v) Open distal femur fractures - Gustilo Anderson type 3C

Protocol

**Figure 1:**
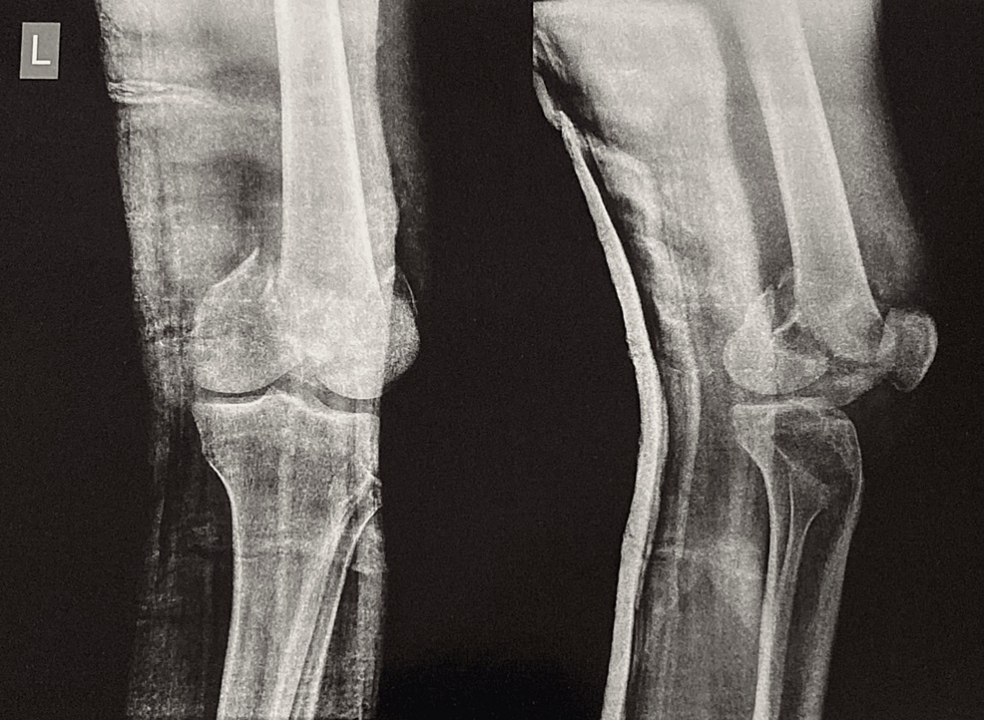
Preoperative radiograph of the knee showing distal femur fracture in anteroposterior and lateral view

On admission, thorough history, general examination, systemic examination, and vitals were recorded in order to rule out any co-morbidities and to establish the nature and mode of injury. All patients were assessed and resuscitated as per Advanced Trauma Life Support® (ATLS®) protocol. Plain radiographs were obtained of the knee and femur of the affected side (anteroposterior and lateral views as seen in Figure 1) as well as routine trauma series radiographs, which included pelvis with both hips, cervical, and lumbar radiographs. 

Above knee slab was applied to immobilise the affected limb with adequate elevation and cold compression. Preoperative investigations including blood investigations, chest radiographs, and electro-cardiograph as per requirements for surgical fitness for anaesthesia were done. CT was done in order to classify the fracture, determine the nature of fragments, plan the surgical approach, determine the choice of implant, and strategise the technique of reduction and fixation. Patients were posted immediately after surgical fitness from anaesthesia. Written and informed consents for the surgical procedure were taken. Injection cefuroxime 1.5 g was given half an hour prior to induction. Xylocaine sensitivity was done on the morning of surgery. Patients who had closed fractures were taken up for surgery within three to four days after admission.

Patients with open fractures were primarily treated for the open wound and a knee-spanning external fixator. A 6L saline wash was given followed by debridement, when needed, and primary suturing was done on admission. The patients were given a course of injectable cephalosporin, metrogyl, and aminoglycosides for five days. Routine betadine sterile dressings were done and the patients were taken up for surgery as soon as the wound condition improved.

Patient were kept in a supine position over a radiolucent operating table, with knee frame utilised to facilitate flexion of the knee during fracture fixation. An image intensifier was positioned on the opposite side of the injured limb. A bolster was utilized under the hip of the affected side when access was needed to the posterolateral aspect of the femur. A high tourniquet was utilized. Painting and draping were done. The surgeon and assistant staff occupied the side of the affected limb. 

**Figure 2:**
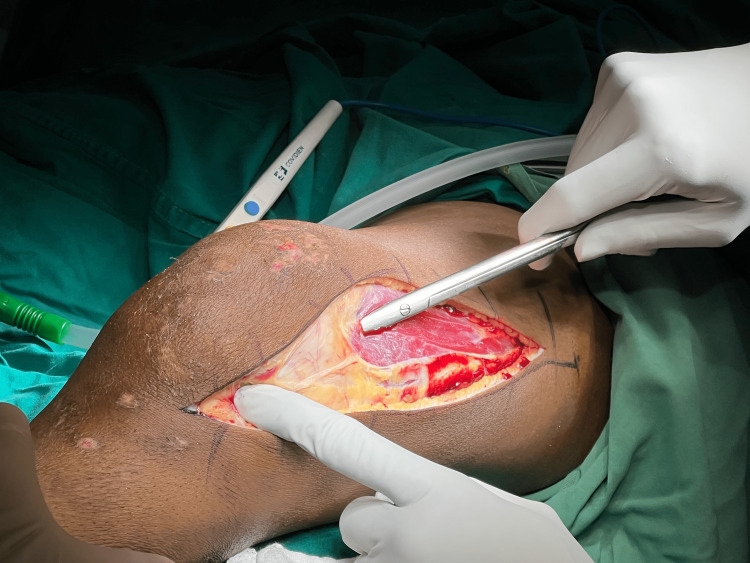
Intraoperative image of approach to distal femur using Swashbuckler approach

The Swashbuckler approach is used to access the distal femur (Figure 2). With the patient in supine as described above, an antero-medial incision is given over the knee. Dissection is continued through the quadriceps fascia, iliotibial band is retracted. Lateral intermuscular septum is detached from vastus lateralis, and quadriceps is pulled medially, and the patella is dislocated medially, allowing adequate access to the distal femur [20].

**Figure 3:**
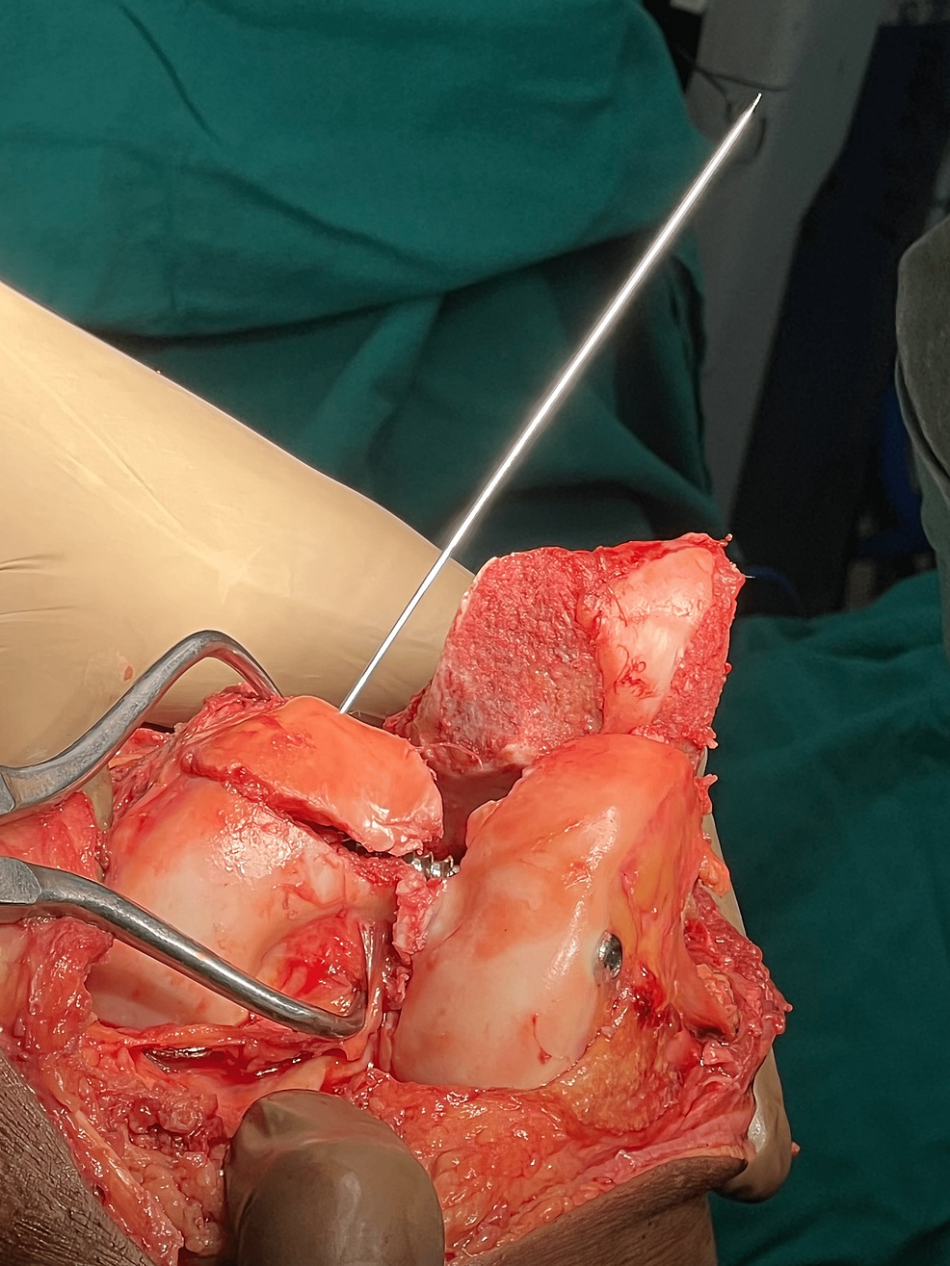
Intraoperative image of reduction of fragments using cannulated cancellous screws to reconstruct the articular segment

**Figure 4:**
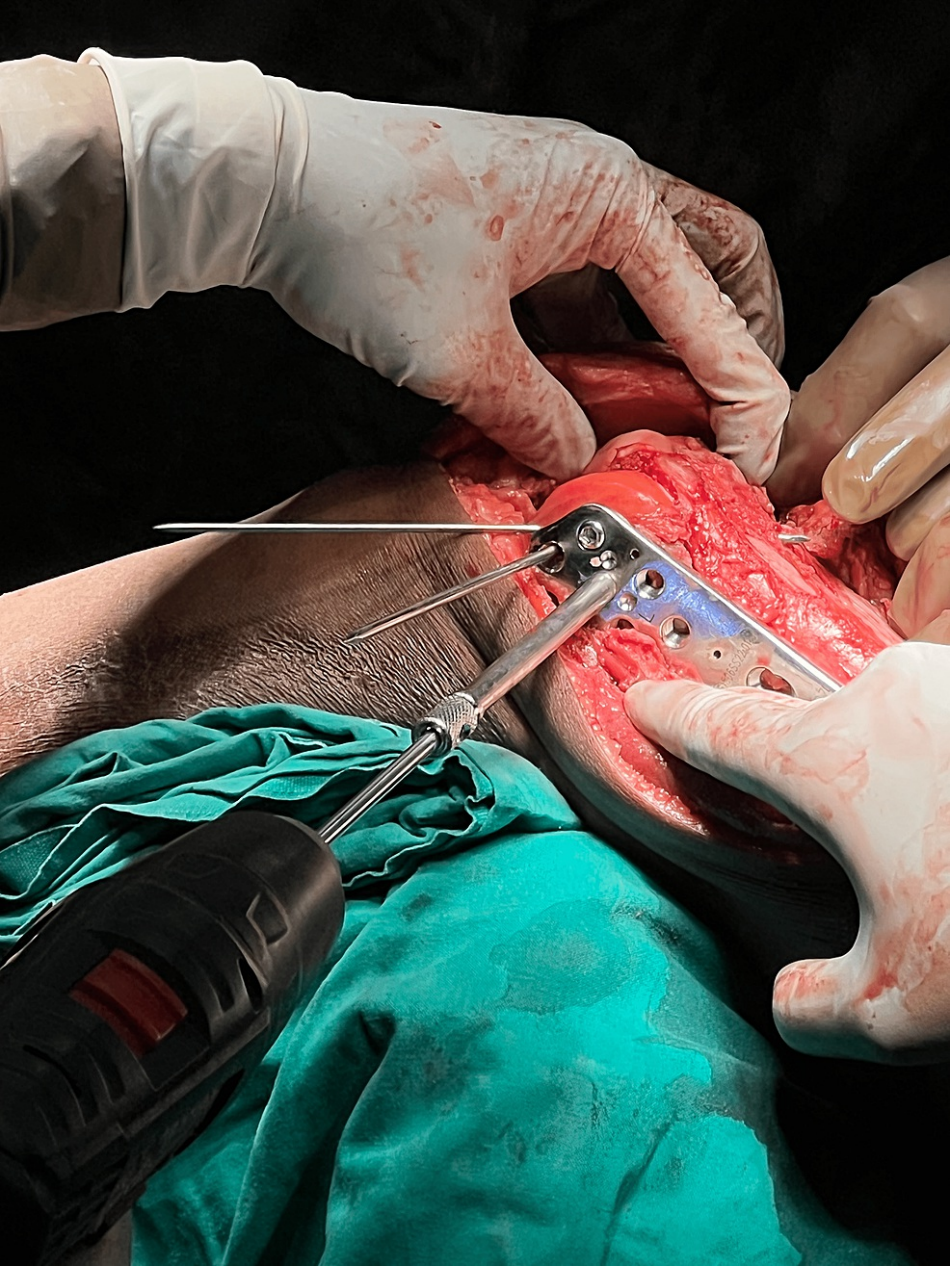
Intraoperative image showing use of lateral distal femur locking plate

**Figure 5:**
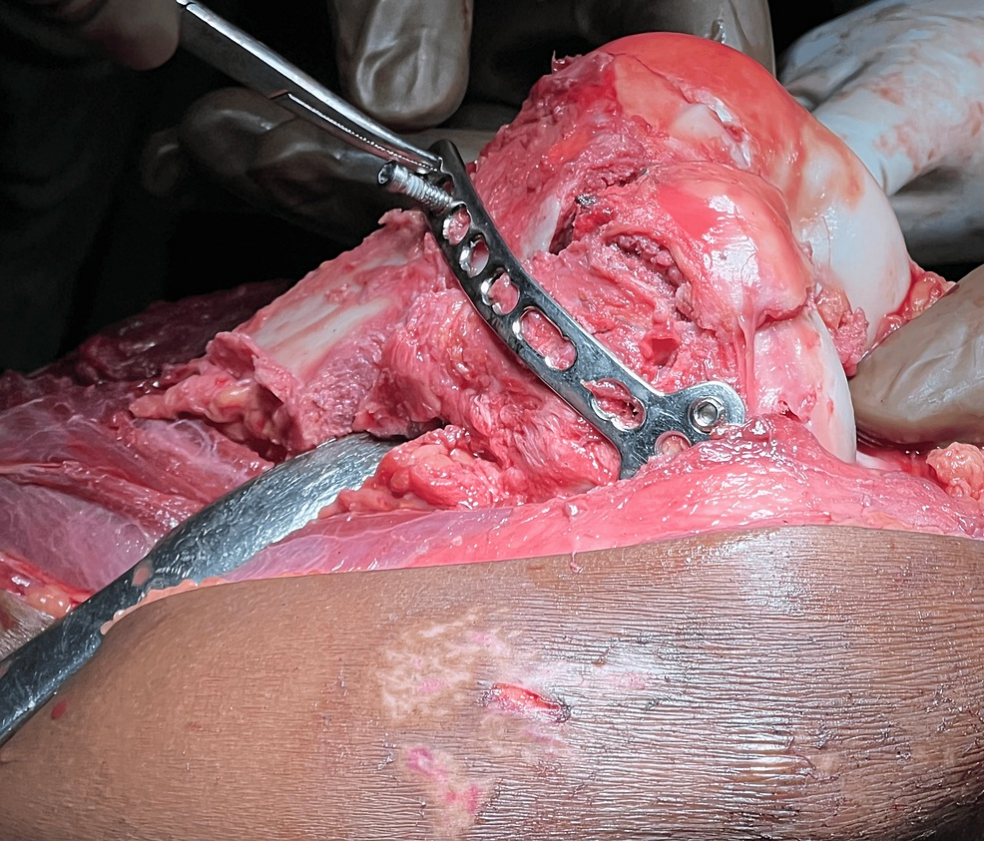
Intraoperative image showing use of medial plate (T buttress plate) for the medial aspect of distal femur

Reconstructing the articular section to anatomical reduction is done using reduction clamps, Kirschner wires, and then fixed using inter-fragmentary screws. Osteochondral fragments can be fixed using headless screws if larger than 5 mm; fragments lesser than 5 mm can be excised. After articular reconstruction (as shown in Figure 3), the articular block is fixed with the metaphysis using a distal femur lateral locking plate (Figure 4). Bridge plating is done if there is comminution in the metaphysis, and the gap is filled with bone graft. Alignment and length are carefully restored. Medial plating is done through the same incision using a T buttress plate or reconstruction plate, depending on the fracture fragment (Figure 5). 

**Figure 6:**
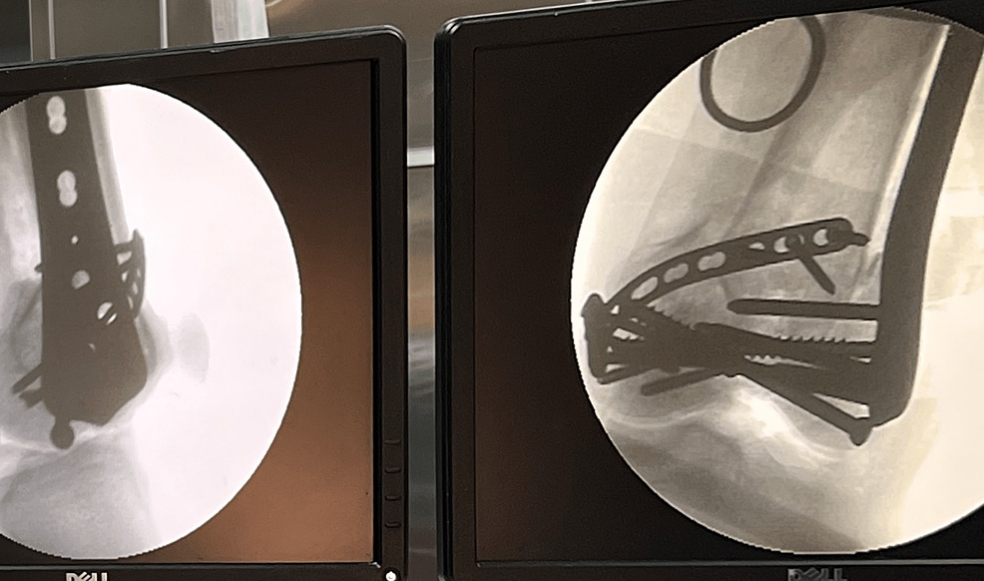
Intraoperative C-arm image to confirm reduction of the fracture

**Figure 7:**
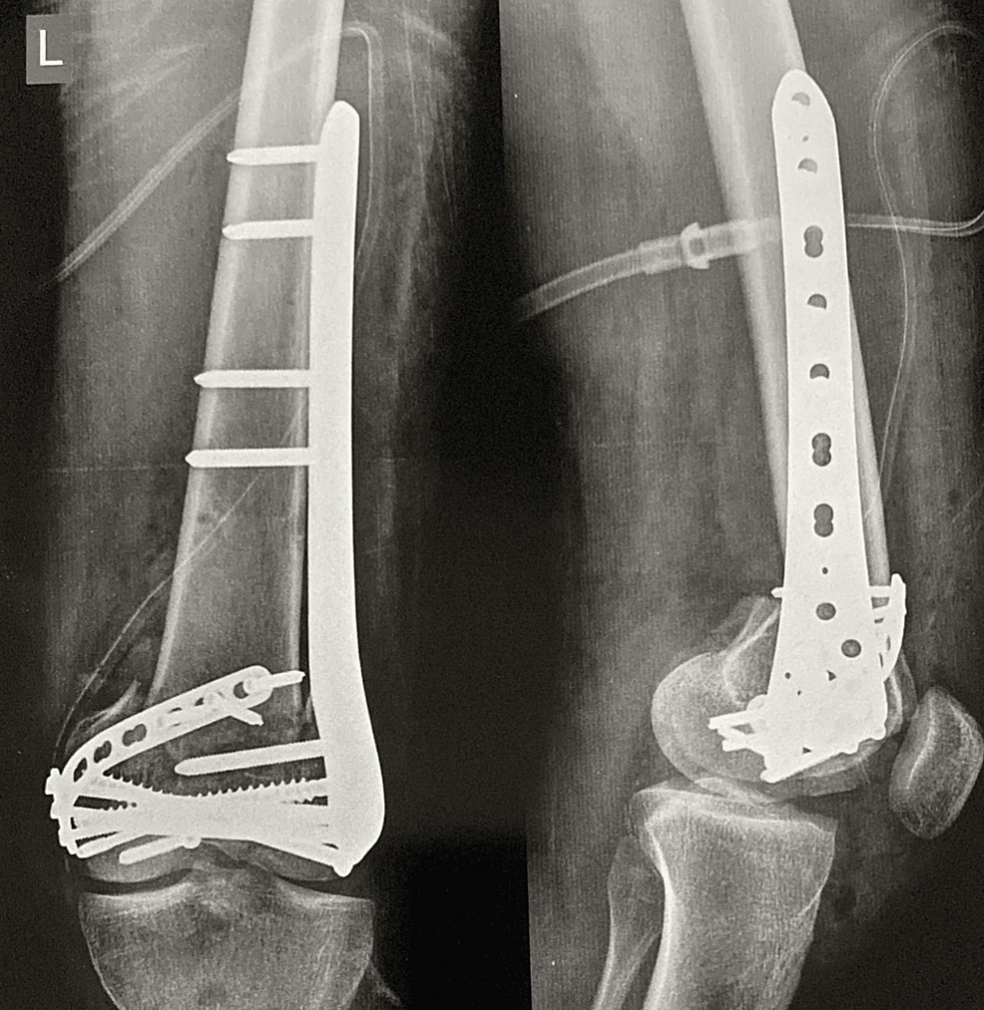
Postoperative radiograph after fixation of the fracture with distal femur locking plate over the lateral aspect and T-buttress plate over the medial aspect

After wound closure, the range of motion of the knee is reassessed and helps in releasing any soft tissue that may have been impinged during the reduction and fixation of fracture fragments. Intra-operative C-arm images are obtained to confirm the reduction of fragments (Figure 6). Postoperative radiograph of the knee in anteroposterior and lateral views is shown in Figure 7.

A robust postoperative protocol is essential for a better clinical and functional outcome. Intravenous antibiotic cover, consisting of cefuroxime 1.5 g with amikacin 750 mg, along with analgesia, and deep venous thrombosis (DVT) prophylaxis was given. Rehabilitation was tailored to suit individual cases and depends primarily on fixation stability. Drain, if used, is removed 48 hours postoperatively. Isometric exercises (static quadriceps and hamstring exercises) are started on the first day, and passive knee range of motion is initiated on the second day. Up to 45 degrees of flexion within the first seven days and gradually increased to around 90-110 degrees by 8-12 weeks. Sutures were removed by the 14th to 18th postoperative day. Plain radiographs were taken of the operated knee, anteroposterior and lateral views, immediately after surgery. Patients were followed up regularly after discharge on day 10, six weeks, and three months to assess the functional outcome, complications, and radiological union. 

Patients would be made to bear 50% weight on the operated limb by the sixth week postoperatively, with aid, and unassisted 100% weight bearing by the 9th-10th week postoperatively. Postoperative knee stiffness was defined by a decrease in range of motion, which would decrease walking capacity to the extent that the patient would require aid to do so [21,22]. Fracture union was defined clinically when the patient was able to weight bear without walking aids, and radiologically union when there is progressive callous formation across two cortices in both views. The follow-up of patients was done by assessing clinically and functionally by Neer's criteria [23] after surgery along with Kolmert scoring [24] three months postoperatively. 

## Results

In this study, 64% of the cases were in the age group of 21-40 years, 24% in the 41-60 years age group, and just 12% in the 61-80 years group (Table 1). The mean age was 39 years.

**Table 1:** Age distribution in the study

Age Group	Count of Age	Percentage
21-40	32	64%
41-60	12	24%
61-80	3	12%
Grand Total	50	

In our study, 76% of the patients were male, and 24% were female (Table 2).

**Table 2:** Gender distribution among patients

Sex	Number of Cases	Percentage
Male	38	76%
Female	12	24%

Sixty percent of the cases were due to road traffic accidents while 40% were due to domestic falls (Table 3). 

**Table 3:** Distribution of cases based on mechanism of injury RTA: road traffic accident

Injury Mechanism	Count	Percentage
RTA	30	60%
Domestic Fall	20	40%

AO (Arbeitsgemeinschaft für Osteosynthesefragen) classification was used in this study. There were no A1-type cases. The majority of the fractures belonged to C2 type (40%, 20 cases). C3 accounted for 20% of the cases. A2, A3, and B1 were the least common, accounting for just 4% of the cases each (Table 4).

**Table 4:** Distribution of cases based on AO Classification of the fracture AO: Arbeitsgemeinschaft für Osteosynthesefragen, the predecessor of the AO Foundation

AO Fracture Classification	Number of cases	Percentage
A1	0	0%
A2	2	4%
A3	2	4%
B1	2	4%
B2	4	8%
B3	6	12%
C1	4	8%
C2	20	40%
C3	10	20%

Out of 50 cases, 44 cases were closed type of fractures, accounting for 88% of the cases. Open fractures accounted for only 12% of the cases (Table 5). 

**Table 5:** Distribution of cases based on the nature of fracture

Nature of Fracture	Number of Cases	Percentage
Closed Fracture	44	88%
Open Fracture	6	12%

As shown in Table 6, T buttress plate was utilized in 38 cases, accounting for 76% of the total cases, and locking recon plate was used for the remaining 12 cases. 

**Table 6:** Distribution of cases based on the medial plate used

Medial Plate Used	Number of Cases	Percentage
T Buttress Plate	38	76%
Locking Recon Plate	12	24%

The majority of the cases did not have any complications, accounting for 84% of the cases. Four cases had stiffness postoperatively, accounting for 8% of the cases, and there were only two cases of non-union and infection each, accounting for 4% of cases, respectively (Table 7).

**Table 7:** Distribution of cases based on postoperative complication

Complication	Number of Cases	Percentage
Stiffness	4	8%
Non-union	2	4%
Infection	2	4%
None	42	84%

Forty-two cases had no FFD. Four cases had FFD of 5 degrees accounting for 8%; meanwhile, there were two cases of 10 degrees and 15 degrees FFD, respectively, accounting for 4% of cases each (Table 8). 

**Table 8:** Case distribution based on fixed flexion deformity

Fixed Flexion Deformity	Number of Cases	Percentage
0 Degrees	42	84%
5 Degrees	4	8%
10 Degrees	2	4%
15 Degrees	2	4%

When maximum flexion was assessed postoperatively in the knee, 12 cases, i.e. 24% of cases, had full flexion up to 130 degrees. Thirty-four cases had flexion up to 120 degrees, accounting for 48% of cases. There was only 4%, i.e. two cases each of flexion up to 100 and 110 degrees (Table 9).

**Table 9:** Distribution of cases based on maximum flexion achieved at the operated knee joint

Maximum Flexion at the knee joint	Number of Patients	Percentage
Up to 120 degrees	34	68%
Up to 130 degrees	12	24%
Up to 110 degrees	2	4%
Up to 100 degrees	2	4%

Out of 50 cases, 24% of cases, i.e. six patients, had excellent outcomes as per Kolmert’s and Neer's scores. Twenty-two patients (44%) had good outcomes, eight cases of fair and poor outcomes each, accounting for 16% of total cases, respectively (Table 10).

**Table 10:** Distribution of cases based on Kolmert and Neer's score

Outcome	Number of cases as per Kolmert Score	Number of cases as per Neer’s Score	Percentage
Excellent	12	12	24%
Good	22	22	44%
Fair	8	8	16%
Poor	8	8	16%

Forty-two cases had normal walking capacity postoperatively, accounting for 84% of cases. Only four cases had restricted walking, accounting for 8% of cases. Meanwhile, four cases required aided walking, accounting for 8% of cases, as shown in Table 11.

**Table 11:** Distribution of cases based on postoperative walking capacity

Walking Capacity	Number of Cases	Percentage
Normal	42	84%
Restricted	4	8%
Aided	4	8%

Postoperative shortening of the operated limb was assessed. Twenty-eight cases had a shortening of less than 0.5 cm, accounting for 64% of the cases (Table 12). Four cases had 0.6-1 cm shortening, accounting for 8% of cases. Ten cases had a shortening of 1.1-1.5 cm, contributing to 20% of cases. Eight cases had a shortening of 1.6-3.5cm, i.e. 16% of the cases.

**Table 12:** Distribution of cases based on postoperative limb shortening

Limb Shortening	Number of cases	Percentage
0.0-0.5 cm	28	64%
0.6-1.0 cm	4	8%
​​​​​​​​​​​​​​1.0-1.5 cm	10	20%
1.6-3.5 cm	8	16%

In this study, 24 cases had postoperative fracture displacement of less than 0.5 cm (which was observed by measuring the fracture displacement from the immediate postoperative and follow-up radiographs), accounting for 48% of cases. Sixteen cases had a postoperative displacement of 1.1-1.5 cm, accounting for 32% of cases. Eight cases had 1.6-2.5 cm fracture displacement. Only 4% of cases had a displacement of 0.6-1 cm (Table 13).

**Table 13:** Distribution of cases based on postoperative fracture displacement

Postoperative Fracture Displacement	Number of Cases	Percentage
0.0-0.5 cm	24	48%
0.6-1.0 cm	2	4%
1.1-1.5 cm	16	32%
1.5-2.5 cm	8	16%

## Discussion

Fractures of the distal femur are challenging due to the proximity to the knee joint and the effect of deforming forces, which needs to be countered during reduction and fixation. Utilization of appropriate and effective reduction techniques is vital for achieving desirable clinical and functional outcomes, which would involve the preservation of vascularity and soft tissue in the vicinity of the fracture. Due to the predominance of high-velocity injuries in distal femur fracture, comminution of the fracture is common and proves to be a major deciding factor when it comes to the implementation of an appropriate method of reduction and fixation and would also dictate the need for bone grafting depending on the bone loss present. Dual plating, also referred to as MAP, in distal femur fractures, has been implemented in the treatment to address the above-mentioned challenges. Dual plating achieves a more rigid and anatomical fixation due to the nature of the stabilisation. 

This study was done to determine the clinical and functional outcome of dual plating of fractures of distal femur. A total of 50 patients with fractures of distal femur underwent fixation with dual plating during the time frame from August 2020 to September 2022. In our study, 76% of the patients were male and 24% were female with 38 and 12 cases, respectively. In the study by Bai et al., the female-male ratio was 1:1. In the study by Rekha et al., 70% of the cases were male with 30% being female [25], which is a ratio comparable to our study. 

The observed average age of patients with distal femur fracture was 39, with the majority of the cases being between 21 and 40 years of age, accounting for 64% of the total cases, supporting that the majority of the cases are that of high-energy trauma in a younger population. The average age documented in the study by Kregor et al. was 49 [26], while in the study by Yeap et al., it was 44 [27]. RTA accounted for 60% of the total cases, therefore contributing to the majority of the cases, while falls contributed to the remaining 40% of the cases. In the study by Rekha et al., 73% of the cases were due to RTA and 27% were due to domestic falls [25]. 

In our study, AO type C2 was the most common pattern of fracture, accounting for 40% of the cases, followed by type C3, which accounted for 20%, while types A2 and B1 were the least common, accounting for only 4% of the cases each. Hence, 68% of our cases were of AO type C fracture pattern, while Kregor et al. noted 50% of their cases were AO type C [26]. In the study by Rekha et al., 46.7% of their cases were AO type C [25].

The majority of the fractures, i.e., 88% of the cases, were closed fractures in nature, while only 12% were open in the current study. The study by Apostolou et al. noted that 20% of their cases were open fractures, with the remaining cases (80%) being closed fractures, which is comparable to our study [28]. Although 84% of cases (n=42) did not have any postoperative complications on follow-up after three months, 8% (four cases) of cases had knee stiffness, 4% (two cases) had non-union, and 4% (two cases) had an infection. Schutz et al. noted 7% of cases had infection postoperatively [29], while Kregor et al. noted 5% infection in his study [26]. In the study by Garg et al, 10% of cases had superficial infections, non-union, and knee stiffness each [30]. 

Only 16% of cases, that is eight cases, had fixed flexion deformity, with four of them having up to 5 degrees. Sixty-eight percent of cases had knee flexion up to 120 degrees while 24% of cases had a full range of motion, i.e. up to 130 degrees of knee flexion. Only 8% of cases had flexion less than 110 degrees, with the least being up to 100 degrees, which was still within functional limits. Our results were comparable to the study by Markmiller et al., where the average range of motion was 0-110 degrees [31]. Garg et al., in their study, had a mean range of flexion up to 124 degrees [30].

Twenty-four percent of cases had excellent outcomes as per Neer’s and Kolmert’s scoring systems, while 44% of cases had good outcomes. Only 16% of cases had fair and poor outcomes. In the study by Garg et al., 50% of cases had excellent outcomes as per Neer’s criteria, 30% had good outcomes, while fair and poor outcomes were 10% each [30]. In this study, 72% of cases had shortening of affected limbs less than 1 cm postoperatively, while only 16% of cases had more than 1.6 cm shortening. In the study by Rekha et al., 10% of cases had a shortening of more than 1.5 cm [25]. Eighty-four percent of cases had postoperative fracture displacement of less than 1.5 cm, which can be co-related with the better outcome of walking. Sixteen percent of cases had displacement of more than 1.6 cm. Park et al., in their study, reported a postoperative mean displacement of 5.6 mm [18]. 

## Conclusions

This study was done on 50 patients with fractures of the distal end of femur. Patients underwent dual plating, i.e. MAP, and the clinical results and functional outcomes were studied. In our study, incidence of knee stiffness was minimal. The majority of our cases had good to excellent outcomes as per Neer's and Kolmert's scoring. Postoperative fracture displacement and limb shortening were minimal. Most of our cases had normal postoperative walking capacity. From the study, we have concluded that outcomes were better for fractures of distal femur when treated with dual fixation, probably due to superior fixation and earlier postoperative mobilization.
